# Antibodies against gonadotropin-releasing hormone (GnRH) and destruction of enteric neurons in 3 patients suffering from gastrointestinal dysfunction

**DOI:** 10.1186/1471-230X-10-48

**Published:** 2010-05-20

**Authors:** Bodil Ohlsson, Eva Ekblad, Béla Veress, Agneta Montgomery, Sabina Janciauskiene

**Affiliations:** 1 Department of Clinical Sciences, Gastroenterology Division, Skåne University Hospital, Malmö, Lund University, Lund, Sweden; 2 Department of Experimental Medical Science, Section for Neurogastroenterology, BMC B11, Lund University, Lund, Sweden; 3 Department of Pathology, Skåne University Hospital, Malmö, Lund University, Lund, Sweden; 4 Department of Surgery, Skåne University Hospital, Malmö, Lund University, Lund, Sweden; 5 Department of Pulmonology, Hannover Medical School, Hannover, Germany

## Abstract

**Background:**

Antibodies against gonadotropin-releasing hormone (GnRH) and gastrointestinal dysmotility have been found after treatment with GnRH analogues. The aim of this study was to examine the presence of such antibodies in patients with dysmotility not subjected to GnRH treatment and study the anti-GnRH antibody effect on enteric neurons viability *in vitro*.

**Methods:**

Plasma and sera from 3 patients suffering from either enteric dysmotility, irritable bowel syndrome (IBS) or gastroparesis were analysed for C-reactive protein (CRP), and for GnRH antibodies and soluble CD40 by ELISA methods. Primary cultures of small intestinal myenteric neurons were prepared from rats. Neuronal survival was determined after the addition of sera either from the patients with dysmotility, from healthy blood donors, antiserum raised against GnRH or the GnRH analogue buserelin. Only for case 1 a full-thickness bowel wall biopsy was available for immunohistochemical analysis.

**Results:**

All 3 patients expressed antibodies against GnRH. The antibody titer correlated to the levels of CD40 (*r*_s _= 1.000, p < 0.01), but not to CRP. Serum from case 3 with highest anti-GnRH antibody titer, and serum concentrations of sCD40 and CRP, when added to cultured rat myenteric neurons caused remarkable cell death. In contrast, serum from cases 1 and 2 having lower anti-GnRH antibody titer and lower sCD40 levels had no significant effect. Importantly, commercial antibodies against GnRH showed no effect on neuron viability whereas buserelin exerted a protective effect. The full-thickness biopsy from the bowel wall of case 1 showed ganglioneuritis and decrease of GnRH and GnRH receptor.

**Conclusion:**

Autoantibodies against GnRH can be detected independently on treatment of GnRH analogue. Whether the generation of the antibody is directly linked to neuron degeneration and chronic gastrointestinal symptoms in patients with intestinal dysmotility, remains to be answered.

## Background

Gastrointestinal motility requires coordination between the intrinsic and the extrinsic nervous systems, the interstitial cells of Cajal (ICCs) and smooth muscle cells [1,2]. The etiology of dysmotility is in most cases unknown, but autoimmunity or inflammation has been suggested. The CD40 pathway is a key mediator for inflammation, and is a marker for the active stage of some autoimmune diseases [3,4].

We have recently described a patient treated with the gonadotropin-releasing hormone (GnRH) analogue buserelin who developed antibodies against GnRH with ensuing degenerative neuropathy including GnRH-containing enteric neurons [5]. Healthy blood donors who served as controls did not have such antibodies [5]. Another GnRH analogue, leuprolide acetate, has been shown to stimulate intestinal motor activity in hypophysectomised and gonadectomised rats [6,7]. The same analogue has in previous studies diminished the symptoms of nausea, vomiting and abdominal pain in irritable bowel syndrome (IBS) [8,9].

This gave rise to the hypothesis that GnRH antibodies may be involved also in idiopathic dysmotility diseases. We therefore examined the expression of such antibodies in sera from patients with gastrointestinal dysfunction and found titers of antibodies in some patients.

The aim of this study was to further examine and describe 3 patients suffering from severe nausea, vomiting and abdominal pain, who had never been treated with any GnRH analogues, but had nevertheless still acquired very high titers of antibodies against GnRH, correlating to soluble CD40 (sCD40) levels, and also had gastrointestinal signs and symptoms.

## Methods

The subjects were treated according to the Helsinki declaration and animals were used in accordance with the European Communities Council Directive (86/609/EEC) and the Swedish Animal Welfare Act (SFS 1988:534). The studies were approved by the Ethics Committee and the Animal Ethics Committee, Lund/Malmö, respectively. Written informed consent was obtained from the patients.

### Study Design

Blood samples were taken from patients on their initial appointment at the Department of Gastroenterology. Serum and plasma were separated and frozen at -20°. Serum was analysed for anti-GnRH antibodies and sCD40, and plasma for C-reactive protein (CRP). Serum was further tested for its capacity to influence neuronal survival of rat myenteric neurons in culture. Case 1 underwent a laparoscopy and histopathological examination was performed on a full-thickness wall biopsy from the ileum.

### Case 1

A 20-year old man was admitted because of nausea, vomiting and severe abdominal pain, accompanied by weight loss. Besides recidivating relapses of herpes infections in the mouth and throat, he suffered no other diseases. The symptoms started at the age of 13 years, when he had a sudden debut of abdominal pain and a collapse. Since then, he suffered occasional periods of abdominal pain and hard stools, alternating with periods of diarrhoea. The results of tests on blood samples taken repeatedly were all within the normal range. The diagnosis IBS was set according to the Rome-II criteria [10]. Both his mother and aunt suffered from functional dyspepsia since several years; else there is no history of hereditary factors.

At the age of 18 years, the symptoms grew worse. The most pronounced symptoms were nausea and abdominal pain, accompanied by weight loss. The symptoms were more and more accelerating, and the patient had difficulties to manage his work and daily life. Vomiting was present some time a week. Blood samples taken repeatedly were all within normal ranges, inclusive cobalamines and folate. Glutenintolerance was ruled out by negative antibodies against endomysium and transglutaminase. Feces haemoglobin was negative, suggesting intact mucosa. Esophago-gastro-duodenoscopy (EGD) showed normal findings in the upper gastrointestinal tract. Treatment with histamine receptor blockers and proton pump inhibitors did not have any impact on the nausea.

As the routine examinations turned out normally, the patient was referred to a psychiatric clinic for treatment. He visited the psychologist regularly for support. However, this treatment did not affect his nausea. Because the symptoms grew worse with abdominal pain, nausea, impaired appetite and weight loss, and altered bowel habits with constipation-dominance, he was admitted to the Department of Gastroenterology.

The dopamine receptor antagonist metoclopramide was then tested without any effect against the nausea. The muscle relaxantive drug papaverin had a small effect on abdominal pain. Further investigation with gastric emptying scintigraphy performed after ingestion of a meal, consisting of egg and bread, showed an emptying rate within the highest field of the reference value. After 60 min, 55% of the content remained in the ventricle (T50 for healthy controls; 40 ± 28 min) [11].

As it was impossible for him to keep weight, also the 5-HT_3_- antagonist ondansetron and the neurolepticum prochlorperazini were tested without any result. Finally, the dopamine receptor antagonist domperidone was prescribed with good result, and the possibility of food intake was slightly improved. As the symptoms aggravated, and could not solely be explained by gastro paresis, the patient was considered for a diagnostic laparoscopy at the age of 23 years. This showed no macroscopic pathology of the small intestine with a normal peristalsis. There were no signs of adhesions, Meckel's diverticulum or tumours. A diamond-shaped, full-thickness wall biopsy was taken one meter proximal to the ileocecal valve and was immediately transported in a wet compress for pathological examination. The histopathological examination showed ganglioneuritis with neuron degeneration. The diagnosis enteric dysmotility was established based on the clinical picture and the histopathology [12].

### Case 2

A 56-year old woman, married, but without children, was admitted because of hard stools, with several days between stool emptying followed by diarrhoea. She also suffered from abdominal bloating and pain, with intermittent colic-attacks and nausea. Apart from analgesics, she was taking no other medication. The symptoms started at the age of 46 years in the form of swallowing disturbances. She was examined with X-ray of the esophagus because of these disturbances. This examination showed quite normal findings. Three years later the procedure was repeated since the symptoms grew worse. This showed a slightly reduced motility of the most distal part of the esophagus. Five years later this was repeated and also included X-ray of the hypopharynx and ventricle. This examination showed normal oral and pharyngeal function. However, the esophagus had a normal transport function, but a tendency to disrupted primary peristalsis. The ventricular emptying was within normal range. Altogether, there were signs of a slightly disturbed esophageal function after some years of symptoms. Examinations with EGD and colon X-ray were normal. She got a retirement at the age of 50 years because of headache, muscle pain and tiredness. Primary Sjögren's syndrome was suspected because of a feeling of dryness in mouth in combination with swallowing disturbances, but serological markers of this disease were negative. Besides analgesics, she had not taken any special drugs. In addition, around the age of 50 years she was suffering from hard stool, with several days in between stool emptying. After the initial stool passage she got diarrhea. She also suffered from a lot of gases and abdominal pain, with intermittent colic-attacks of pain. Metoclopramide had some effect on the swallowing disturbances, but the patient felt best when avoiding food intake. Motility stimulating laxantia was prescribed for her constipation.

After still 4-5 years the symptoms grew worse and she was referred to the Department of Gastroenterology. The symptoms fulfilled the Rome-II criteria for IBS [10] and she had already gone through several examinations, why no further investigations were performed. Metoclopramide was later seponated because of cardiac arrhythmia. The symptoms were not of such a magnitude that a full-thickness biopsy was considered. Further, the patient had attacks of cardiac arrhythmia which made anaesthesia undesirable.

### Case 3

A 72-year old man was admitted because of gastro paresis and diarrhea, refractory to treatment. At the age of 1.5 years, he was sick from diabetes mellitus. He aquired most of the secondary complications with nephropathy, neuropathy and macroangiopathy with ensuing cardiovascular diseases. Around the age of 30 years, he started to suffering from diarrhea and abdominal pain. Ultra sound examinations of the abdomen performed several times during the years were all normal. He was operated on with nephrectomy for ureter malignancy at the age of 50 years. Postoperatively he received cytostatica in the form of 3 periods of methotrexate, vinblastine and CCNU, and radiation therapy. By-pass operation was performed at the age of 60 years. Through the years different immunological tests were performed, e.g. antibodies against thyreoid, adrenal glands and gliadin. At the age of 62 years, an extensive examination was performed to finally find out the etiology to the abdominal symptoms. Tests for examination of bacterial overgrowth, bile salt malabsorption and fat malabsorption were all within normal ranges. EGD revealed a light reddish mucosa. Celiaki was ruled out trough duodenal biopsies. Enterography revealed a dilatation and reduced motility of duodenum and jejunum. On an initial colon X-ray a polyp was identified. Computer tomography showed a cystic change in caput pancreatis. Gastric emptying scintigraphy showed retention where 75% of the content remained in the ventricle after 1 h. The diagnosis gastro paresis was given. A second colon X-ray performed 2 years later revealed diverticulosis. Different pharmacological treatment regimes were tested through the years, i.e. erythromycine, metoclopramide, cisapride, domperidone and fibers. Metronidazole and tetracycline were tested towards bacterial overgrowth. No one of the drugs had any overt effect. The symptoms escalated and an EGD was performed 8 years later which showed patchy reddish mucosa of the ventricle. Eradication of Helicobacter pylori did not influence the symptoms. He was eventually referred to the Department of Gastroenterology because of nausea, loss of appetite, early satiety, abdominal fullness and bloating and vomiting. He also suffered from imperative diarrhea. He had then had peritoneal dialysis for 3 years. The patient was deceased soon afterwards.

### Antibodies

Analysis of anti-GnRH antibodies was carried out by an ELISA method as earlier described in detail [5]. The absorbance at 405 nm (A_405_) was measured in a Titertek Multiscan at 1:40 dilution. Serum from 20 healthy blood donors served as controls.

### CD40 and CRP

CD40 was measured by an ELISA kit developed for quantitative detection of sCD40 in human body fluids (Alexis^© ^Biochemical, Bender MedSystems, Vienna, Austria). The detection limit is 7.92 pg/ml, and the intra-assay coefficient of variation has been calculated to 5.5%. CRP was analysed by a routine assay at the Department of Clinical Chemistry, Skåne University Hospital, Malmö.

### Cell culture

Primary cultures of small intestinal myenteric neurons were prepared from adult female rats (n = 25), using a modification of previously described methods [13,14]. The rats were anaesthetised, the small intestine was exposed and longitudinal muscle with attached myenteric ganglia were stripped and incubated in Ca^2+ ^- and Mg^2+ ^- free Hanks' balanced salt solution (HBSS) containing collagenase II (1.5 mg/ml), protease (1.5 mg/ml), trypsin (1.25 mg/ml) and EDTA (0.01%), triturated and vortexed. The cell suspension was centrifuged (74 g, 7 minutes), washed in HBSS and diluted to 2 ml in Neurobasal A (NBA), supplemented with 10% FCS, 0.5 mM glutamine, 50 U penicillin and 50 μg streptomycin sulphate per ml. Cultures were prepared by seeding 50 μl of the cell suspension together with 450 μl of NBA to each well (69 mm^2^) in 8-well, uncoated Tissue Culture slides (BD Falcon) and grown in an incubator (37°C, 5% CO_2_). After 4 days *in vitro *(DIV) 400 μl of the medium was replaced by fresh medium and either sera or the GnRH analogue was added (see below).

Neuronal survival was determined by cell counting after the addition of serum from the 3 cases and 3 healthy blood donors (dilution 1:50 -1:200), antiserum against GnRH raised in rabbit (anti-LH-RH; PROGEN Biotechnik GmbH, Heidelberg, Germany; dilution 1:50-1:200) or buserelin (10^-8^-10^-6 ^M) (Suprefact^©^, Sanofi-Aventis, Solna, Sweden). The agents were present for 2 days in the cultures. At the end of this experimentation period the cultures were fixed for 30 minutes in a mixture of 2% formaldehyde and 0.2% picric acid in 0.1 M phosphate-buffer, pH 7.2, followed by rinsing twice in Tyrode's solution containing 10% sucrose, and frozen until processed for immunocytochemistry. Parallel controls were cultured in NBA (supplemented as described above). As general neuronal marker, antibodies against human protein gene product 9.5 (code no PGP9.5; Ultraclone, Isle of Wight, UK; dilution 1:1600; [14]) were used. The surviving neurons on the entire glass cover slip were counted.

### Histopathology

From the full-thickness biopsy of case 1, two transversal, full-thickness slices perpendicular to each other were cut and embedded in paraffin for conventional transversal sections. The remaining, larger part of the biopsy was embedded *in toto *for tangential sectioning, which makes it possible to examine large areas of the myenteric plexus. Serial sections from all the blocks were stained according to a protocol for chronic intestinal pseudo-obstruction (CIPO) analysis including a variety of antibodies [5,15]. In addition, immunostainings for herpes virus type 1 and 2 were also performed, based upon the anamnestic data of previous herpes infection.

**Figure 1:**
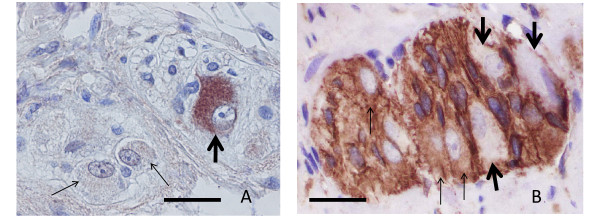
**Control myenteric ganglia**. A: one GnRH positive neuron (thick arrow) and two GnRH negative neurons within the ganglion. (GnRH immunohistochemistry; bar: 20 μm). B: three GnRH receptor positive neurons (thin arrows) and three receptor negative nerve cells (thick arrows) in control ganglion. (GnRH receptor immunohistochemistry; bar: 20 μm).

Furthermore, anti-GnRH antibodies (anti-LH-RH; PROGEN Biotechnik GmbH, Heidelberg, Germany) were applied to sections at 1:75 dilution following previous staining for optimising the dilution (Figure 1A). The specificity of the immunostaining was examined through absorption of the antibody by addition of the GnRH peptide (Suprefact^©^, Sanofi-Aventis, Bromma, Sweden) before applying the solution to the sections. For the staining of the GnRH receptor, anti-GnRH receptor antibody (anti-LH-RH receptor; Sigma-Aldrich, Stockholm, Sweden) was applied to sections at 1:100 dilution after similar tests as with anti-GnRH antibody (Figure 1B). Negative controls by replacing the antibody were always performed together with the immunostainings. The number of GnRH or receptor +/- neurons was counted. The number of ICCs was determined by counting ICC-nuclei both within the intermyenteric plexus and within the circular muscle. As controls for GnRH and GnRH receptor +/- neurons and the number of ICCs, sections from 11 cases of bowel resection due to small intestinal non-obliterating carcinoma with normal macro- and microscopic appearance 10 cm above the tumour were used. The criteria for enteric ganglioneuritis were described previously [15].

### Statistical methods

Histological values are expressed as mean ± standard deviation (SD). Spearman's test was used to calculate correlations. Neuronal survival in cell culture was calculated and expressed as a percentage (mean ± standard error of the mean; SEM) of the control run in parallel. Statistical differences were determined using one-way analysis of variance test (ANOVA) followed by Bonferroni's *post hoc *test. P < 0.05 was considered statistically significant.

## Results

### GnRH Antibodies

**Figure 2:**
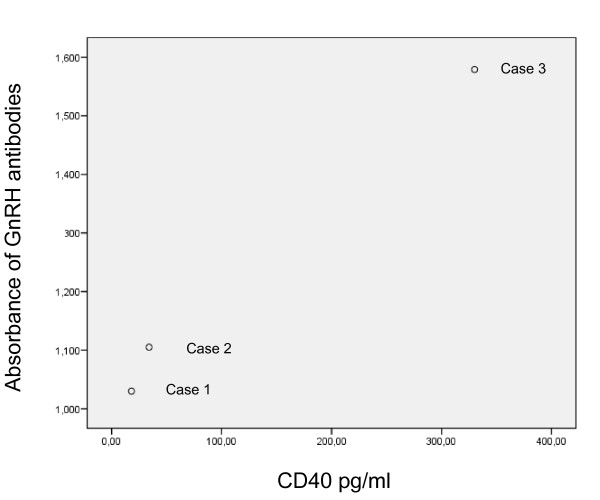
**The correlation between the concentration of antibodies against gonadotropin releasing hormone (GnRH) at 1:40 dilution and soluble CD40 in serum (pg/ml), *r*_*s *_= 1.000, p < 0.01**. The measured absorbance at 405 nm of different plasma samples using the ELISA procedure described was transformed to the corresponding rabbit anti-GnRH with the aid of the standard curve. The sample was considered as positive if the absorbance was > 0.800.

All 3 cases had titers of antibodies against GnRH (Figure 2). None of the controls had any antibodies (cut off: 0.800 optical densities).

### CD40 and CRP

The concentration of sCD40 correlated positively to the levels of GnRH antibodies (Figure 2), whereas CRP (2, 2 and 29 mg/L, respectively) did not correlate either to sCD40 or to antibodies (*r*_*s *_= 0.866, p < 0.33 for both).

### Cell culture

**Figure 3:**
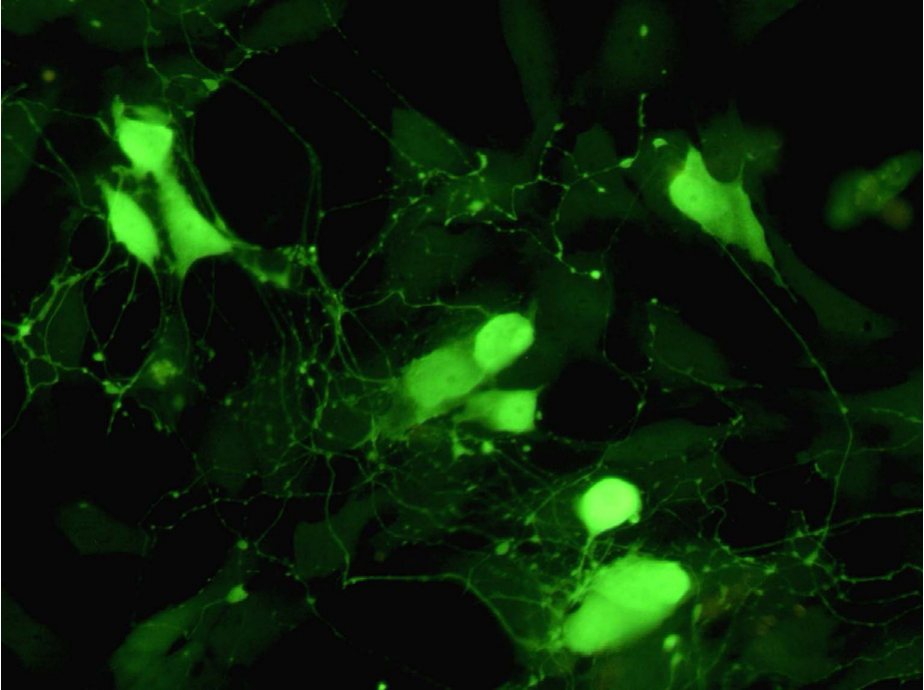
**Immunocytochemical staining with PGP of myenteric neurons grown for 6 days**. The cultured neurons survive well; they group into ganglion-like structures and grow a prominent arborising network of nerve terminals. Bar 40 μm.

Myenteric neurons were seeded and grown for 4 days before the addition of the sera or buserelin. The density of myenteric neurons at 4 DIV was 8.9 ± 0.5/mm^2 ^(n = 14). The cultured myenteric neurons stained well with antibodies against PGP 9.5 and were thus easily distinguishable (Figure 3). The neurons were uniformly dispersed throughout the culture well. They often grouped into clusters containing 4-12 neurons and possessed a rich network of arborising fibers, frequently connecting other neurons.

**Figure 4:**
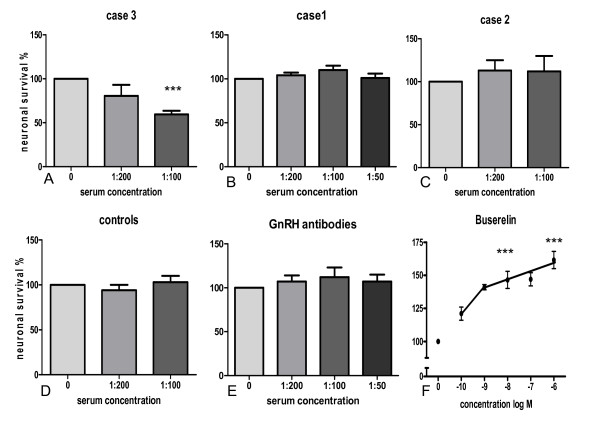
**Neuronal survival of neurons pre-cultured for 4 days followed by 2 days of culture in the presence of (A) serum from case 3 (n = 5), (B) serum from case 1 (n = 8), (C) serum from case 2 (n = 3), (D) serum from healthy blood donors (n = 8), (E) gonadotropin-releasing hormone (GnRH) antibodies raised in rabbit (n = 6-7) and (F) buserelin (n = 8)**. Addition of sera from cases number one and two, from healthy blood donors or GnRH antibodies caused no change in neuronal survival while serum from case 3 significantly and concentration-dependently reduced neuronal survival. Buserelin markedly enhanced neuronal survival in a concentration-dependent manner. *** p < 0.01, as compared to the controls run in parallel.

Addition of serum from case 3 to cultures of myenteric neurons significantly and concentration-dependently reduced neuronal survival; at the highest concentration (1:100) 59.4 ± 4.2% of the neurons survived as compared to controls run in parallel in the absence of the serum. Addition of sera from case 1 and 2 or control sera from 3 different, healthy blood donors did not affect neuronal survival. The results are summarised in Figures 4.A-D.

The presence of antiserum against GnRH raised in rabbit did not affect the survival of myenteric neurons in culture (Figure 4.E). The presence of buserelin resulted in a concentration-dependent increase in neuronal survival as compared to neurons cultured in medium without buserelin (Figure 4.F). The highest buserelin concentration tested resulted in a markedly enhanced neuronal survival, 168 ± 13% as compared to controls run in parallel (100%).

### Histopathology

Case 1 was examined by a full-thickness biopsy. The mucosa, submucosa, and smooth muscle cells showed no pathologic alterations. Within the *submucous ganglia *the number of GnRH+ neurons was diminished to 23% compared to 72 ± 8% in the controls.

**Figure 5:**
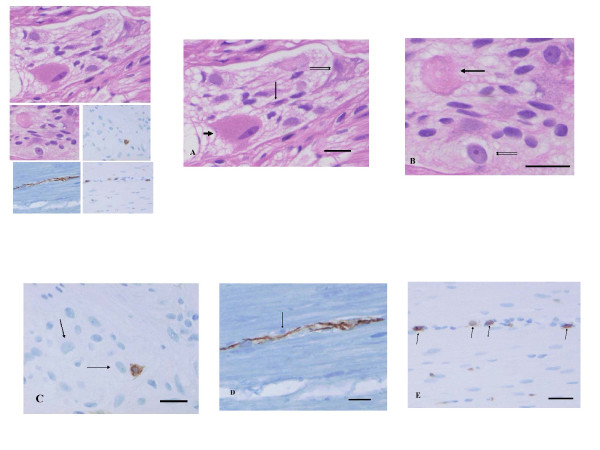
**Details of a myenteric ganglion and surrounding muscle tissue**. 5A. The cytoplasm of the enlarged neuron is filled with small tightly packed eosinophilic granules, the nucleus is compressed, elongated (large arrow). There are two other neurons with vacuolized cytoplasm (double arrows) and another small, shrunken neuron (short arrow) (Haematoxylin & eosin;bar: 20 μm). 5B. Eosinophilic residual body with shadows of vacuoles of a necrotic neuron (large arrow). The double arrows show a degenerating neuron containing very large nucleolus and extremely pale cytoplasm with a few perinuclear vacuoles. (Haematoxylin & eosin; bar: 20 μm). 5C. Ten μm large, activated T-lymphocytes in the vicinity of two neurons (arrows). (CD3 immunohistochemistry; bar: 20 μm). 5D and E show detail of the circular muscle with a small nerve as followed up on serial sections. 5D. There is a small cell containing a densely stained rounded nucleus (arrow) along the small nerve. (Neurofilament immunohistochemistry; bar: 20 μm). 5E. Several CD3+ T-lymphocytes are seen along the nerve shown on 5D. (CD3 immunohistochemistry; bar: 20 μm).

Many *myenteric neurons *were swollen (>50 μm), pale and often vacuolised showing chromatolysis; whereas a few enlarged neurons had an "oxyphilic-appearance" (Figure 5A). "Hyaline bodies" were observed within and at the ganglia (Figure 5B). The counting of GnRH+/- neurons revealed 28% positive myenteric neurons in the patient versus 62 ± 10% in the controls. The immunostaining for GnRH receptor was positive in 26% myenteric neurons in the patient compared to 46 ± 6% in the controls. Intraneural CD3+ T-lymphocytes were observed both within the large nerve bundles and the myenteric ganglia close to neurons (Figure 5C). About 90% of these T-cells were CD8+. Some of the small intra-muscular nerves showed axon vacuolation with CD3+/8+ lymphocytes along these nerves (Figure 5D-E). The immunostainings for herpes type 1 and 2 were negative. Based upon these features, the pathological diagnosis was ganglioneuritis, combined with decrease of GnRH and GnRH receptor positive neurons.

The number of ICCs in the circular muscles was slightly increased: 19 ICCs/1 mm^2 ^muscle versus 14 in controls, whereas it was normal in the intermyenteric plexus. The ICCs showed normal cytomorphology.

## Discussion

These 3 cases had in common symptoms of nausea, vomiting and abdominal pain and all had antibodies against GnRH in serum, although none had received GnRH or its analogues. Notably, histopathological examination of case 1 showed ganglioneuritis and neuron degeneration combined with a decrease in the number of GnRH and GnRH receptor positive neurons in the full-thickness ileum biopsy. Case 1 had relapses of herpes simplex infections, and it has been demonstrated in rats that herpes simplex viruses are stored in myenteric ganglia of the gastrointestinal tract [16]. A connection between cytomegaloviruses and the development of CIPO by direct viral infection and inflammation of the myenteric ganglia has been described [17]. Therefore, one could suggest that viral damage to the nerves leads to exposure of GnRH and to the development of antibodies against GnRH [18]. In the present study, at the time of the biopsy, no herpes-viral antigens could be detected in the bowel wall and there was no histological sign of cytomegalovirus infection.

It is important to notify, that there was a difference regarding the histological findings between case 1 and the previously reported woman who developed GnRH antibodies after buserelin treatment and *in vitro *fertilization (IVF) [5]. The latter developed degeneration of the myenteric neurons and severe loss of GnRH positive neurons without lymphocytic ganglioneuritis [5], while case 1 had ganglioneuritis. This may be explained either by the fact that she was biopsied in an earlier stage of the process than case 1, or by missing the foci of ganglioneuritis in the previous reported case due to uneven and focal distribution of the lymphocytic attack on the myenteric ganglia. In healthy controls, there was described a higher amount of GnRH than GnRH receptors. This may depend on down-regulation of the receptor due to stimulation. This discrepancy was not seen in the case.

Previous studies have described the presence of autoantibodies against neuronal structures in CIPO, especially as secondary to malignancy [19], but this is the first time that such antibodies have been found in patients suffering from IBS or diabetes mellitus. A new classification of dysmotility diseases based upon the etiology has been discussed instead of the symptomatology as today [12]. Although the patients of the present report expressed different clinical findings, there may be a common etiological factor on the molecular basis, leading to different clinical expression. We have earlier described the presence of GnRH in the human enteric nervous system (ENS) [5]. In rats, GnRH and GnRH receptor mRNA has been found in the myenteric plexus [20,21]. The effect on the ENS is not completely evaluated, but GnRH has been shown to inhibit the release of gastric secretions and gastrin release in dog [22], to stimulate motor function in the gastrointestinal tract in female rats [6,7] and to restore motor function in a patient suffering from CIPO [17].

The reason for the development of GnRH antibodies and their biological role in the 3 studied cases is not known. Indeed, in all cases the titer of anti-GnRH antibodies correlated to the plasma level of sCD40, which is related to the CD40 expression and the activity of the CD40-CD40L signalling pathway. CD40 is a 45-50 kDa type I transmembrane protein which belongs to the TNF receptor superfamily and it is expressed on the surface of numerous immune and non-immune cells. The CD40 is an important signalling pathway in the initiation and maintenance of immune responses, including those associated with chronic inflammatory diseases [3]. For instance, in autoimmune diseases such as Grave's disease and Hashimoto's thyreoiditis, CD40 seems to be a marker of the active stage of the diseases [4]. Increased plasma levels of sCD40 and their correlation with the extent of mucosal inflammation have been recently reported in patients with inflammatory bowel disease [23].

Based on the finding that the titers of anti-GnRH antibodies correlate with sCD40 levels, we hypothesized that in our studied cases GnRH antibodies may be related to neuronal damage. To test the hypothesis that anti-GnRH antibodies can directly cause neuronal damage, we cultured rat myenteric neurons in primary cell culture and measured cell viability after 2 days exposure to serum from cases 1, 2 and 3. Case 3 was in an active inflammatory stage reflected by high anti-GnRH antibody titer and high plasma concentrations of sCD40 and CRP, and addition of serum from case 3 to cultured rat myenteric neurons caused remarkable cell death. In contrast, serum from cases 1 and 2 having lower anti-GnRH antibody titer and lower sCD40 levels had no significant effect on neuronal viability. Sera from other diabetic patients, with or without gastroparesis and acute inflammation, but without GnRH antibodies, did not destroy cultured myenteric neurons (unpublished data).

To in further detail study the effect of anti-GnRH antibody on neuronal viability, we exposed neuronal cultures to various concentrations of purified commercial anti-GnRH antibody. However, under our experimental conditions, anti-GnRH antibody *per se *showed no effect on neuronal viability. The finding that buserelin, a long-acting GnRH analogue, increased neuronal viability was really unexpected which since in the pituitary, GnRH and its agonists act as functional antagonists when administered continuously [24]. Similar desensitization may also occur in other GnRH target cells. Thus, increased neuronal survival in this system may very well have occurred in the presence of downregulated GnRH receptor-coupled signaling pathways. This latter result also leads to speculation that reduction of the GnRH concentration due to autoantibody generation against GnRH in the bowel wall may initiate neuron degeneration.

Indeed, interpretation of our data in terms of a biological role on anti-GnRH is somewhat complicated by the fact that serum from cases 1 and 2 did not induce neuronal death *in vitro*. Currently, one can only speculate that anti-GnRH toxicity may be dependent on antibody heterogeneity, as described, for example, in atherosclerosis [25] or other factors, in addition to auto antibodies, are necessary to be present to amplify or execute neurodegenerative effects. On the other hand, the GnRH antibodies may be secondary to neuron degeneration and subsequent exposure of neuron material to inflammatory cells.

The answer on these questions would be a significant step forward in better understanding pathology of gastrointestinal dysmotility.

## Conclusion

Here we have described the presence of autoantibodies against GnRH and reduction of GnRH and its receptor in enteric neurons in one patient with gastrointestinal complaints. Further, serum from one patient with antibodies and acute inflammation destroyed rat neurons in culture at the same time as GnRH exerted a neuron-protective effect. The associations between these different findings have to be settled. Development of autoantibodies against GnRH may be related to neuron degeneration and gastrointestinal symptoms in a subgroup of patients, but this warrants further research.

## Competing interests

The authors declare that they have no competing interests.

## Authors' contributions

BO designed the study, collected the data, paid the study and wrote the manuscript. EE designed, performed and paid for the studies on the cultured neurons. BV evaluated the full-thickness biopsy, AM performed the laparoscopy and SJ evaluated the ELISA results. All authors contributed to the manuscript with constructive criticism, and read and approved the final manuscript.

## Pre-publication history

The pre-publication history for this paper can be accessed here:

http://www.biomedcentral.com/1471-230X/10/48/prepub
